# Alleged Cure of Cancer

**Published:** 1855-10

**Authors:** 


### ALLEGED CURE OF CANCER.

M. Landolfi, chief surgeon of the Neapolitan army, has of late attracted much attention in Italy and Germany, having seemingly cured cancer by means of the topical use of chloride of bromine, in combination with several other chlorides. This success has produced such sensation as to induce the Emperor of the French to give the author six beds at the Salpetriere Hospital (where the insane and aged of the female sex are admitted), to afford him a fair opportunity of proving the efficacy of the remedy. This is the right, practical, and sensible way of dealing with such questions. We are in the meanwhile bound to state that *L'Union Medicale*, of May 1, 1855, contains a letter from M. Leriche, of Lyons, who says that he has strong doubts about the actual *bona fide* cancer having ever been cured by the above-named application, as he has steadily used the chloride of bromine for ten months, in various cases of cancer, without having succeeded in warding off the fatal issue of this formidable disease.

The formula of the paste used by M. Landolfi, is the following; Chloride of bromine, three parts; chloride of zinc, two parts; chloride of antimony, one part: chloride of gold, one part; powder of liquorice sufficient to make into a paste. The principal agent is the chloride of bromine, which has lately been used by itself. Cancers of the skin, the epithelial variety, lupus, &c., are treated by a combination of chloride of bromine with basilicon ointment. M. Landolfi's view is to change a malignant ulceration into a simple one. For this purpose, he formerly left a piece of linen spread with the paste as long as a fortnight upon the part, but now he uses imbricated pieces of lint, similarly spread, and leaves them only twenty-four hours. The surrounding parts are protected by an ointment composed of one drachm of chloroform to an ounce of axunge. The author considers that the chloride of bromine acts not only topically, but that the specific is absorbed and aids the cure. Hence he gives, as an adjuvant, a certain number of pills which contain a minute proportion of the chloride. When the pledgets spread with caustic paste are taken off, after the above-mentioned twenty-four hours, a line of demarcation is observed which separates the altered from the healthy tissues. Bread poultices are then applied, or else lettuce leaves, or basilicon ointment, which should be changed every three hours, until the eschar is thrown off, which event takes place from the eighth to the fifteenth day.

Numerous experiments have been made in Italy and Germany, and some successful cases have been recorded. It should be added, that there is much fairness about the proceedings; M. Landolfi does not choose the cases, is anxious to make the remedy extensively known, and publishes the unfavorable as well as the favorable results. The Committee appointed in Paris to report respecting the experiments at the Salpetriere, is composed of the physicians of the hospital, Drs. Moissenet, Cazalis, and Manec, assisted by MM. Mounier, Broca, and Furnari. This report will be an important document, as the diagnosis will no doubt be very carefully made; but the question of recurrence can only be solved by investigations spreading over several years.—*Medical News.*

